# Correcting for Verbal Autopsy Misclassification Bias in Cause-Specific Mortality Estimates

**DOI:** 10.4269/ajtmh.22-0318

**Published:** 2023-04-10

**Authors:** Jacob Fiksel, Brian Gilbert, Emily Wilson, Henry Kalter, Almamy Kante, Aveika Akum, Dianna Blau, Quique Bassat, Ivalda Macicame, Eduardo Samo Gudo, Robert Black, Scott Zeger, Agbessi Amouzou, Abhirup Datta

**Affiliations:** 1 Department of Biostatistics, Epidemiology and Informatics, University of Pennsylvania, Philadelphia, Pennsylvania;; 2 Department of Biostatistics, Johns Hopkins University, Baltimore, Maryland;; 3 Department of International Health, Johns Hopkins University, Baltimore, Maryland;; 4 Center for Global Health, Centers for Disease Control and Prevention, Atlanta, Georgia;; 5 ISGlobal, Hospital Clínic - Universitat de Barcelona, Barcelona, Spain;; 6 Centro de Investigação em Saúde de Manhiça (CISM), Maputo, Mozambique;; 7 ICREA, Barcelona, Spain;; 8 Pediatrics Department, Hospital Sant Joan de Déu, Universitat de Barcelona, Barcelona, Spain;; 9 Consorcio de Investigación Biomédica en Red de Epidemiología y Salud Pública (CIBERESP), Madrid, Spain;; 10 Instituto Nacional de Saúde (INS), Maputo, Mozambique

## Abstract

Verbal autopsies (VAs) are extensively used to determine cause of death (COD) in many low- and middle-income countries. However, COD determination from VA can be inaccurate. Computer coded verbal autopsy (CCVA) algorithms used for this task are imperfect and misclassify COD for a large proportion of deaths. If not accounted for, this misclassification leads to biased estimates of cause-specific mortality fractions (CSMFs), a critical piece in health-policy making. Recent work has demonstrated that the knowledge of the CCVA misclassification rates can be used to calibrate raw VA-based CSMF estimates to account for the misclassification bias. In this manuscript, we review the current practices and issues with raw COD predictions from CCVA algorithms and provide a complete primer on how to use the VA calibration approach with the calibratedVA software to correct for verbal autopsy misclassification bias in cause-specific mortality estimates. We use calibratedVA to obtain CSMFs for child (1–59 months) and neonatal deaths using VA data from the Countrywide Mortality Surveillance for Action project in Mozambique.

## INTRODUCTION

Accurate and credible cause-of-death (COD) data are critical to understand, interpret, and address the burden of diseases and tailor public health policymaking at subnational, national, and regional levels. A complete diagnostic autopsy (CDA) is the gold-standard procedure for determining COD. When full autopsy is not affordable or feasible, medical certification of COD (MCCOD) is often conducted using all medical information relevant to the terminal illness. In low- and middle-income countries (LMICs), CDAs are very rarely conducted due to cultural, religious, and infrastructural constraints, whereas MCCOD has suboptimal coverage that is usually limited to deaths that occur in health facilities and is of variable quality.^1^ For settings without the capacity to conduct CDA or where they are infrequently done, a nonclinical approach called “verbal autopsy” (VA) is commonly used. VA is a systematic postmortem interview of the relatives of the deceased on the health history, signs, and symptoms of the fatal illness that can potentially identify the COD. Although the reliability of the VA at the individual level is questionable,^2^ it is often the only feasible option and has become a key source of COD data in LMICs that do not have fully developed civil registration and vital statistics systems with MCCOD information.^3^ In addition, VA-based results are often useful for studying population-wide trends of cause of death.

There are two ways to assign a COD from a VA report. One practice is to have physicians review the VA (physician-coded VA [PCVA]).^3^ This process is time- and resource intensive, and PCVA results can be inaccurate or hard to standardize across physicians, countries, or regions.^4^ A scalable alternative to PCVA is to use an automated algorithm termed “computer-coded VA” (CCVA) that inputs a VA record and outputs a probable COD. The format of the VA instrument has now been standardized by the WHO and is compatible with many CCVA automated diagnostic algorithms like InterVA,^5^ InSilicoVA,^6^ EAVA,^7^ SmartVA,^8^ the Naive Bayes Classifier,^9^ among others.

The automation afforded by CCVA, offering diagnosis of COD for large databases of VA records, has led to its increased adoption in large-scale VA studies.^10^ The “raw” estimates of cause-specific mortality fractions (CSMFs)—the percentage of deaths attributable to a given cause—are obtained as the proportion of the total number of deaths in the VA database that are predicted to be from that cause by the CCVA algorithm. The CSMF estimates can be stratified by age groups, sex, geographical regions, or other subgroups. CSMF estimates from CCVA algorithms can produce results similar to those from physician review.^11^

However, this widespread practice of aggregating CCVA outputs to obtain CSMFs has ignored the fact that CCVA algorithms are not perfect, and their accuracy depends both on the quality and geographical coverage of the training data and the modeling assumptions used in creating the algorithm. The COD determination from CCVA is not the true COD; it is only a predicted one and is prone to misclassification. Multiple studies have now shown that CCVA-predicted COD (VA-COD) suffers from misclassification bias; for a significant proportion of deaths (often > 50%), the predicted cause from CCVA differs from the cause obtained using more comprehensive information.^12^^–^^14^ The misclassification of CCVA can be assessed by comparing CCVA outputs to medical certification based on COD obtained from CDA, minimally invasive tissue sampling (MITS; also called minimally invasive autopsy, or MIA),^15^^–^^17^ or some “reference standard” combination of laboratory, pathology, and medical imaging results such as used by the Population Health Metrics Research Consortium (PHMRC).^18^

The misclassification bias of CCVA gets propagated into the raw (uncalibrated) estimates of CSMFs based on the CCVA-determined COD. Biased CSMF estimates from CCVA can mislead public health professionals and decision-makers to potentially misallocate the use of resources to prevent mortality. This misclassification is also prevalent for PCVA, which has been shown to perform worse than CCVA at identifying COD at both the individual and the population level in some settings.^4^ In this manuscript, we focus on calibrating CSMFs from CCVA, but the calibration approach can also be applied to CSMFs from PCVA.

Datta et al.^13^ developed a method called “calibratedVA” that calibrates the initial raw CSMF estimate from a CCVA algorithm by adjusting for the misclassification bias of the algorithm. The method requires a paired dataset of COD having both CCVA and a reference standard COD based on more comprehensive medical and laboratory information to learn the misclassification rates of the CCVA algorithms. The misclassification rates are then used to calibrate the raw CSMF estimates in a hierarchical Bayesian modeling framework. Calibration has been shown to substantially improve CSMF estimates over the raw (uncalibrated) estimates from CCVA.^13^^,^^14^

In this manuscript, we offer a statistical primer on how to use calibratedVA to correct for misclassification bias of CCVA algorithms. We provide a complete workflow of the methodology that estimates the raw CSMF and the misclassification rates, combines them to produce calibrated CSMF estimates, and provides data-driven model comparison metrics to compare and choose between the raw and calibrated CSMF estimate. Finally, we discuss how calibratedVA also combines predictions from multiple CCVA algorithms to produce a single CSMF estimate based on an ensemble calibration method. The ensemble method is preferable over the use of a single CCVA algorithm because it guards against incorrect results produced by a poorly performing algorithm. We apply calibratedVA to obtain CSMF estimates for child (aged 1–59 months) and neonatal deaths in Mozambique. The methodology can be used to correct for COD misclassification bias in VA-based projects in other countries.

## MATERIALS AND METHODS

### COMSA Mozambique verbal autopsy data.

We use VA data from the nationally representative Countrywide Mortality Surveillance for Action (COMSA) program in Mozambique to obtain raw (uncalibrated) CSMF estimates. COMSA provides CSMFs at the national and subnational levels for Mozambique based on active surveillance for deaths in 700 clusters of approximately 300 households each, with a total population of 923,031 people. We collected 11,614 VAs on deaths across all age groups that occurred from 2017 to 2021. The majority of deaths that are registered in COMSA occur outside of a hospital and thus are not assigned an official COD. For each registered death in COMSA, a VA is conducted. The dataset used in this analysis includes records for 1,841 deaths of children (1–59 months old) and 818 neonatal deaths from May 2018 to May 2021 from all 11 provinces of Mozambique. The VA questionnaire used for COMSA corresponds to the WHO 2016 VA tool.^1^ The forms have been programmed into the Open Data Kit software^19^ for data collection on a tablet. In-person interviews are conducted with a respondent determined to have been the child’s usual caregiver, which is most often the mother.

### Child Health and Mortality Prevention Surveillance (CHAMPS) Network MITS data.

To estimate the misclassification rates of COD predictions for the CCVA algorithms, we use data from the CHAMPS network. CHAMPS is an ongoing comprehensive child mortality surveillance project that performs MITS to inform determination of COD for children (1–59 months), neonates, and stillbirths at sites across several countries, including Mozambique.^20^ MITS COD assignments in these age groups have been shown to be accurate (∼75% concordance) when compared with the full diagnostic autopsies.^16^^,^^21^

The CHAMPS data used in this manuscript contain records for 426 child (1–59 months) and 614 neonatal deaths that occurred within the CHAMPS network hospitals in Bangladesh, Ethiopia, Kenya, Mali, Mozambique, Sierra Leone, and South Africa, from July 2017 through December 2020. MITS is only conducted for “disease-related” deaths and not for trauma or accidental deaths. The MITS-COD was determined through review of postmortem biopsy pathology and screening tests for a large array of pathogens, as well as medical history and clinical records, by a panel of physicians (including pediatricians), pathologists, microbiologists, and public health specialists. In CHAMPS, the COD report using MITS provides a full chain of events, initiated by the underlying cause, followed by the morbid or antecedent conditions(s), and finalizing with the immediate cause. For each death in CHAMPS, the VA record was also available in addition to the MITS-COD.^17^ Because the CCVA typically only provides the underlying cause of death, to estimate misclassification rates of VA (see “Misclassification bias of CCVA algorithms” below), we pair the “underlying” cause from the MITS-COD with the VA-COD for each of the deaths in the CHAMPS dataset.

### CCVA algorithms and uncalibrated CSMFs.

To obtain the raw (uncalibrated) estimates of age group–specific CSMFs, we use COD diagnosis from two CCVA algorithms, InSilicoVA^6^ and EAVA,^7^ for each COMSA child and neonate record. These CCVA algorithms were used due to their fundamentally different nature of decision-making. InSilicoVA is a probabilistic (Bayesian) method that assigns a COD for a VA record based on the likelihood (probability) of the reported VA responses (illness, signs, and symptoms) for that record given each COD. InSilicoVA is broadly similar to InterVA,^5^ another popular CCVA algorithm, but offers a more statistically principled treatment of the binary (yes/no) and the missing VA responses. Hence, we used InSilicoVA instead of InterVA.

The second CCVA algorithm, EAVA, is not a statistical algorithm. It is based on medical decision-making rules. The approach relies on expert-derived algorithms of VA illness signs and symptoms for each COD and a hierarchy to select the main COD from among all identified comorbidities.^22^ EAVA does not use a probability framework, training dataset, or symptom-given-cause matrix like InterVA or InSilicoVA. Instead, it is a deterministic algorithm and for each death produces a single most likely COD. It is, however, driven by the ordering of causes in the hierarchy of all causes of interest. More details on the implementation of the two algorithms to obtain COD is provided in Supplemental Section 3. Once the specific cause has been determined by InSilicoVA and EAVA for each death, causes are grouped into broader cause categories (see “Aggregation of causes into broad categories” below) to be used for raw estimation or calibration.

For each neonatal and child (1–59 months) death record in the data, we obtained the top (most probable) COD from InSilicoVA. These are then aggregated to obtain the raw (uncalibrated) InSilicoVA CSMFs simply as the proportion of all VA records assigned to be from a given cause. The same procedure is repeated with EAVA to obtain the raw EAVA CSMF. More formally, for an age group and a chosen CCVA algorithm, the raw CSMF estimate for a cause *j* for that age group will be given by

$$\text{Raw CSMF for cause}\;j\;=\;\frac{\text{Number of VA records with CCVA predicted COD as cause}\;j}{\text{Total number of VA records}}$$ (1)

### Misclassification bias of CCVA algorithms.

Misclassification occurs from a CCVA algorithm when the algorithm assigns an individual a COD that is different from that individual’s reference COD (in this case the MITS-COD). Previous work has shown that using the misclassification rates of a VA algorithm to obtain a calibrated CSMF estimate can greatly improve accuracy over the uncalibrated CSMF estimate.^13^ Because the misclassification rates for the CCVA algorithms are not known for COMSA, we use the CHAMPS data to estimate these misclassification rates. For each CHAMPS record we use the MITS-COD paired with the VA-COD. We can estimate the misclassification rate (cause-specific true-positive and false-negative rates) of the CCVA algorithm as described below.

For a cause *i* we calculate the true-positive rate of the CCVA algorithm for that cause as the proportion of CHAMPS deaths with MITS COD *i* that are also assigned to COD *i* by the CCVA algorithm (VA-COD). Similarly, for a pair of causes *i* and *j*, we can calculate the cause pair–specific false-negative rate as the proportion of CHAMPS deaths with MITS-COD *i* that are assigned to COD *j* by the CCVA algorithm (i.e., VA-COD is *j*). We collect these true-positive and false-negative rates in a misclassification rate matrix **M** whose entry **M***_ij_* in the *i*th row and *j*th column is given by:

$$M_{ij}=\frac{\left\{\text{Number of CHAMPS cases with MITS COD cause}\;i,\text{and CCVA predicted COD as cause}\;j\right\}}{\text{Total number of CHAMPS cases with MITS COD cause}\;i}$$ (2)

The diagonal entries of the misclassification matrix are the cause-specific true-positive rates (sensitivities), and higher values would indicate higher accuracy for the CCVA algorithm. The off-diagonal entries of the matrix contain the cause pair–specific false-negative rates and lower values would indicate higher accuracy for the CCVA algorithm. A perfect CCVA algorithm with no misclassification bias would have 1 (100%) on the diagonals and 0 on the off-diagonals of **M**.

### Aggregation of causes into broad categories.

Estimating the misclassification rates of a CCVA algorithm requires estimating all the entries of the misclassification matrix. For *C* many causes, this would imply inferring about *C*^2^ many true-positive or false-negative rates (one corresponding to each cause pair). If we want to use the full set of more than 30 causes, this would mandate estimating the 30 × 30 misclassification rates matrix (i.e., 900 cause pair–specific misclassification rates). Such a task is impossible with only a few hundred MITS deaths (426 for children 1–59 months, 614 for neonates). Hence, to ensure stable estimation of the misclassification rates, we grouped the original larger set of causes into a smaller set of broad cause categories.

For children, we use seven broad causes of death in our study: pneumonia, malaria, diarrhea, severe malnutrition, HIV, other infections, and other causes of death. Other infections in children include meningitis, typhoid fever, and hepatitis. Other causes in children include cancer, injury, and congenital malformation. For neonates, we use five broad causes: congenital malformation, infection, intra-partum related events (IPREs), prematurity, and other. Infection in neonates includes neonatal tetanus, meningitis and encephalitis, diarrhea, pneumonia, and sepsis. The other category for neonates includes causes like injury. These broad causes represent the main causes of death of young children and neonates known from the extensive literature on child mortality in LMICS.^3^^,^^23^

### Correcting for misclassification bias using calibrated VA.

The misclassification matrix of a CCVA algorithm can be used to correct for its misclassification bias in the raw CSMF estimates. The calibration is essentially a back-solving procedure to adjust for the CCVA sensitivities. We elucidate this with a simple hypothetical example. Suppose there are only two causes A and B, and we know that a given CCVA has sensitivities of 95% and 65% for the two causes, respectively. This knowledge about the sensitivity of CCVA may be derived from some paired dataset of VA records and a reference COD (like MITS-COD) from an auxiliary dataset (like the CHAMPS data in this application). Also, suppose that from the unpaired data of only VA records, the uncalibrated CSMFs are 53% for cause A and 47% for cause B. It is evident that these uncalibrated CSMFs are biased. Sensitivity for cause B is only 65%. Therefore, the CCVA mistakenly assigns 100% − 65% = 35% of people who truly die of the cause B to cause A. This will lead to a higher uncalibrated CSMF for the cause A than its true CSMF. We can use these sensitivities to calibrate for the true CSMF *p*_A_ and *p*_B_ = 100% − *p*_A_, respectively, of cause A and B as follows:

$$53\%=p_{\text{A}}*95\%+p_{\text{B}}*(100\%-65\%)$$ (3)

$$47\%=p_{\text{A}}*(100\%-95\%)+p_{\text{B}}*65\%$$ (4)

The above equations allow to calibrate (back-solve) for the unknown CSMFs *p*_A_ and *p*_B._ These calibrated CSMFs are *p*_A_ = 30% and *p*_B_ = 70%, respectively, reflective of the substantial bias in the uncalibrated CSMF.

When more than two causes are being considered, the back-solve is not straightforward, and direct attempts to back-solve this multivariate system of equations may lead to unstable and absurd estimates (estimated cause proportions lying outside of 0–100%). Hence, the calibration approach was formalized into a probability model that avoids both these problems.^13^ The model has two parts for the two data sources: COMSA and CHAMPS. The model for the COMSA data models the raw CSMF as a weighted sum of the calibrated CSMF weighted by the misclassification rates similar to the equations above. The model for the CHAMPS data helps estimate these misclassification rates using Equation (2). The two parts are jointly used in a Bayesian framework that simultaneously estimates both the misclassification matrix and the calibrated CSMFs. Being a Bayesian algorithm, calibratedVA offers both point estimate of the CSMFs as well as 95% credible intervals which are used for inference about changes in CSMF after calibration. The COMSA national sample includes sampling weight to correct for the selection of clusters with probability proportionate to size and oversampling in four provinces. However, for the analysis and description in this paper, only unweighted CSMFs were used. See Supplement Section 1 for a technical overview of the calibration method. The calibratedVA method is made publicly available as a software via Github R-package.^24^ The Github repository contains all the code as open-access and a vignette with example scripts to use the software is also publicly available.^25^

### Ensemble calibration method.

The available CCVA algorithms generally do not agree with each other for a substantial proportion of deaths, and, for a given VA data point, it is challenging to know a priori which CCVA algorithm will be most accurate. Hence, Datta et al.^13^ developed an ensemble calibration approach that uses COD predictions from multiple CCVA algorithms. The ensemble method estimates the misclassification rates of each CCVA algorithm separately and then calibrates by back-solving for the CSMF that agrees best with all the data (i.e., the misclassification rates and the raw CSMFs from each of the CCVA algorithms). The ensemble calibration estimates the misclassification rates of the different algorithms and weights the more accurate ones favorably. Hence, the ensemble calibration has been shown to guard against inadvertent use of a poor performing CCVA algorithm and, therefore, performs better than VA calibration using a single CCVA algorithm. In our analysis, in addition to conducting individual VA calibration with each CCVA algorithm to present the respective calibrated CSMFs, we also implement the ensemble calibration by simultaneously using the predicted COD data from both InSilicoVA and EAVA to produce a unified CSMF estimate. As recommended by Datta et al.,^13^ we present the estimate from the ensemble calibration as our final CSMF estimate for each age group.

We compared the calibrated estimate from each respective CCVA algorithm with the corresponding uncalibrated estimate. For the ensemble method, we compare the calibrated ensemble estimate with the uncalibrated ensemble estimate, which is simply the equally weighted average of the uncalibrated CSMF estimates from the different CCVA algorithms.

### Overview of VA calibration pipeline.

**Figure 1.**
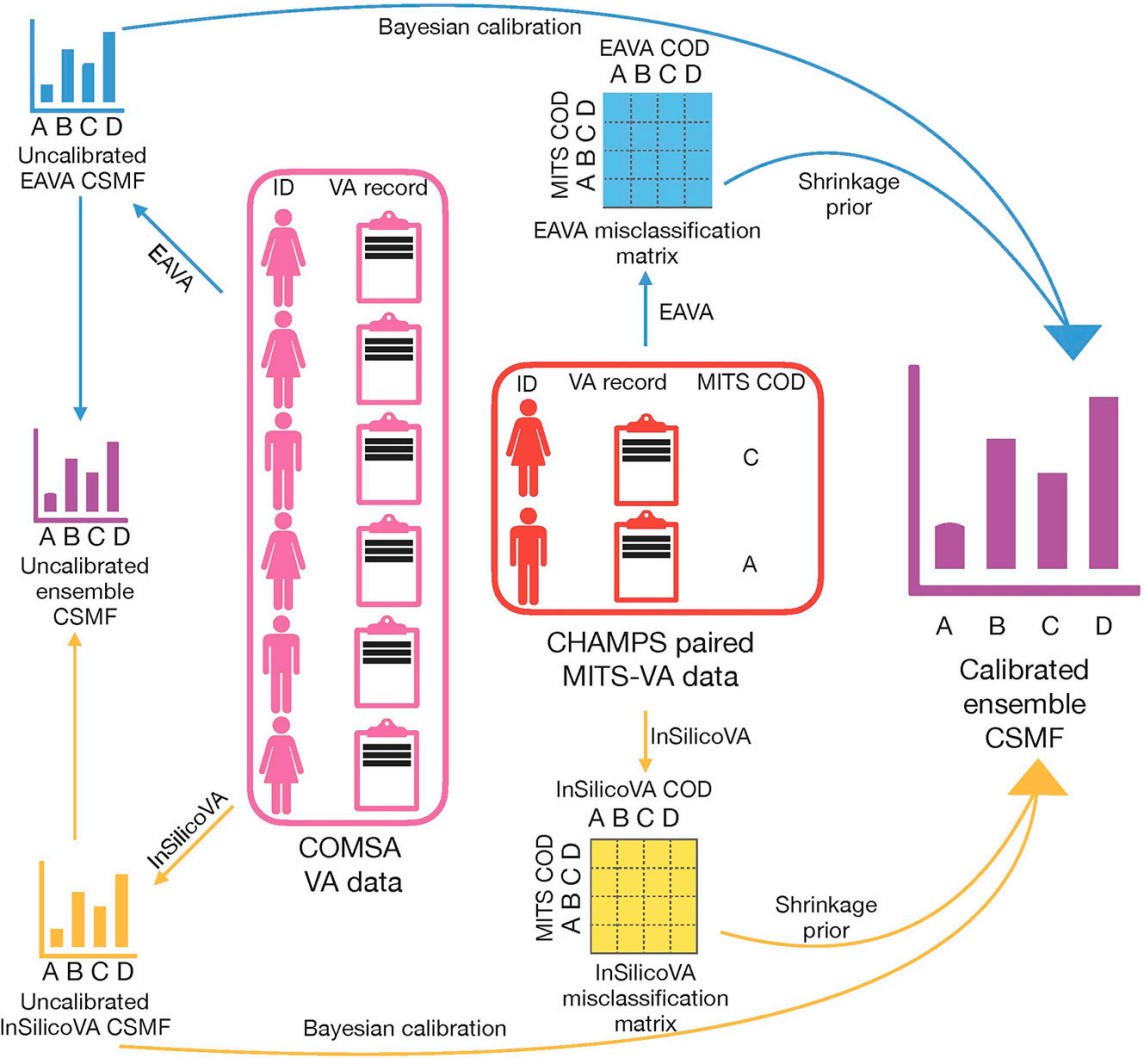
A complete pipeline for calibration of verbal autopsy (VA) based cause specific mortality fractions (CSMF), using minimally invasive tissue sampling (MITS), for Countrywide Mortality Surveillance for Action (COMSA) Mozambique. CHAMPS = Child Health and Mortality Prevention Surveillance; COD = cause of death.

We provide a summary of the entire VA calibration procedure in Figure 1. For each VA record in the dataset (in our case, the COMSA VA dataset), the predicted COD is obtained and aggregated, leading to the raw uncalibrated CSMF estimates. This is repeated for each CCVA algorithm considered (InSilicoVA and EAVA in this analysis), and the resulting CSMFs are averaged to obtain the uncalibrated ensemble estimate. From the CHAMPS data of paired VA-COD and MITS-COD, we obtain the misclassification rates for each CCVA algorithm. We feed both the uncalibrated CSMFs and misclassification rates for both algorithms into the VA calibration pipeline to obtain the ensemble calibrated estimate.

To compare the results from the calibrated models to the uncalibrated CSMFs for each CCVA algorithm (InSilicoVA, EAVA, ensemble), we use the widely applicable information criterion (WAIC).^26^ WAIC is an estimate of a model’s ability to model future data but using only already collected data.^27^ Lower WAIC is better. Details of how the WAIC is calculated are provided in Supplemental Section 2.

## RESULTS

### Child (1–59 months) results.

#### Raw VA summary statistics.

All data analyses were conducted and figures were produced using R software.^28^ We present the summary (predicted counts and distributions) of different cause categories among the COMSA VA data using both InSilicoVA and EAVA. Among the 1,841 child deaths from the dataset, the analysis excludes VA records for 252 child deaths for which the EAVA diagnoses were inconclusive. Table 1 presents these numbers for the remaining 1,589 VA records for under-five child deaths. We see that, according to InSilicoVA, diarrhea and other infections each contributed to nearly one-quarter of the deaths (25%); malaria and pneumonia were also significant causes, both contributing to > 15% of deaths; and severe malnutrition, HIV, and other each contributed to < 10% of the deaths. For EAVA, the distributions showed some difference from the InSilicoVA results. According to EAVA, > 30% of the COMSA child (1–59 months) deaths were attributed to other infections, which stood out as the single largest cause category. Pneumonia (21.5%) and diarrhea (19.4%) were also major categories, whereas deaths attributed to malaria, severe malnutrition, HIV, and other were each < 10%.

**Table 1:** Raw counts and percentages of cause of death in children (1–59 months) as predicted by InSilicoVA and EAVA for COMSA child VA records (*N* = 1,589)

Algorithm	Malaria	Pneumonia	Diarrhea	Severe malnutrition	HIV	Other	Other infections
InSilicoVA, *n* (%)	303 (19.2)	251 (15.8)	399 (25.1)	74 (4.7)	58 (3.7)	128 (8.1)	376 (23.7)
EAVA, *n* (%)	134 (8.4)	342 (21.5)	308 (19.4)	98 (6.2)	125 (7.9)	99 (6.2)	483 (30.4)

COSMA = Countrywide Mortality Surveillance for Action; HIV = human immunodeficiency virus; VA = verbal autopsy.

#### CHAMPS MITS data and VA misclassification rate matrices.

**Figure 2.**
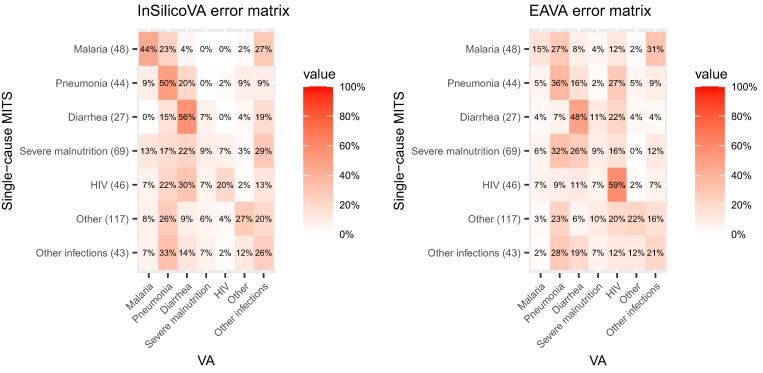
Misclassification rate matrices for InSilicoVA and EAVA for children (1–59 months) based on the Child Health and Mortality Prevention Surveillance minimally invasive tissue sampling (MITS) data. The row totals indicate the counts for MITS underlying cause of death. HIV = human immunodeficiency virus; VA = verbal autopsy.

To evaluate the misclassification rates of the two CCVA algorithms, we used the paired dataset of MITS-COD and VA-COD for child (1–59 months) death records from CHAMPS. Thirty-two child deaths of the CHAMPS/MITS study had inconclusive EAVA diagnoses and were excluded from the analysis. The misclassification rates of CCVA on the MITS data are presented in Figure 2. The diagonal entries of the misclassification matrices are the cause-specific true-positive rates (sensitivities) of the VA-COD agreeing with the MITS-COD, and higher values would indicate higher accuracy for the CCVA algorithm. For example, the entry in the first row, first column of the InSilicoVA misclassification matrix in Figure 2 (left) is 44%. This means that of the deaths for which the MITS-COD was malaria, the VA-COD was also malaria for 44% of them. The off-diagonal entries of the matrices contain the cause pair–specific false-negative rates (i.e., the fraction of cases with a specific MITS-COD that are assigned to a different COD by the CCVA algorithm). Lower values of these off-diagonal entries indicate higher accuracy for the CCVA algorithm. As another example, the first row, second column of the InSilicoVA misclassification matrix in Figure 2 (left) is 23%. This indicates that 23% of the child deaths that were assigned to malaria by MITS were assigned to pneumonia by InSilicoVA.

Both CCVA algorithms have very large misclassification rates with cause-specific sensitivities that are very low (∼10% for severe malnutrition for both algorithms) to moderate (∼60% for the MITS diarrhea deaths for InSilicoVA and the MITS HIV deaths for EAVA). Many of the cause pair–specific false-negative rates are > 20% for either algorithm.

#### Calibrated CSMFs.

**Figure 3.**
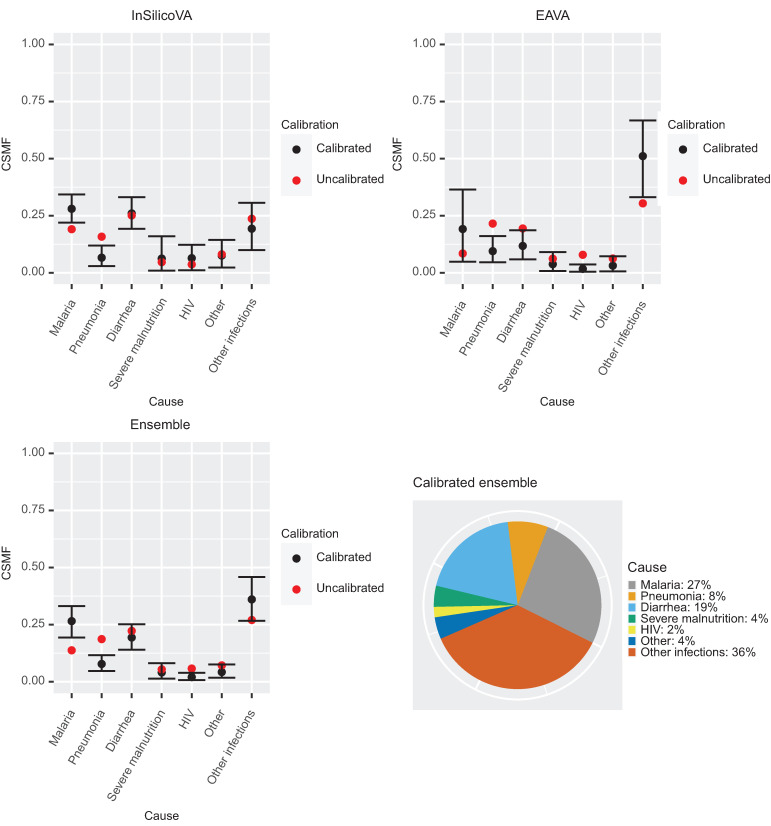
Calibrated cause-specific mortality fraction (CSMF) estimates for children (1–59 months) for the three verbal autopsy (VA) methods (top left: InSilicoVA; top right: EAVA; bottom left: Ensemble). The estimates (black points) are the posterior mean of the calibrated CSMFs. The black bars give the 95% credible intervals. For reference, the uncalibrated CSMF is also plotted (red points). Bottom right is the pie chart of the point estimates (posterior means) of the calibrated ensemble CSMFs. HIV = human immunodeficiency virus.

Figure 3 presents the results of VA-calibration. We present both the uncalibrated and calibrated CSMFs along with the 95% interval estimate for the calibrated CSMF. The exact CSMFs corresponding to these plots are provided in Supplemental Table 1. In addition to results for the individual CSMF algorithms (InSilicoVA and EAVA), we provide the results from the ensemble calibration, which gives the final CSMF estimate.

For InSilicoVA (Figure 3, top left), the main changes after calibration are an increase in CSMF for malaria and a decrease in CSMF for pneumonia. We observe from Figure 2 that a large proportion of MITS-COD for malaria is falsely classified by InSilicoVA as pneumonia or other infections. Because these two causes combined account for 40% of the COMSA deaths according to InSilicoVA (Table 1), the calibration adjusts for this misclassification, thereby resulting in increased proportion of malaria deaths. For pneumonia, many of the deaths caused by MITS-diagnosed malaria, diarrhea, and other infections are falsely classified as pneumonia, whereas among the MITS pneumonia deaths only substantial misclassification is the diagnosis of diarrhea for 20% of deaths. These results imply that there are overall more false positives for pneumonia than false negatives, and hence the CSMF decreases after calibration.

For EAVA, after calibration, the CSMFs for malaria and other infections increase, and those of pneumonia and diarrhea decrease (Figure 3, top right). The reason for the change in the EAVA CSMF of malaria is the same as that for InSilicoVA: substantial undercounting of true malaria deaths by EAVA. For pneumonia, a large percentage of MITS pneumonia cases are misclassified as HIV. Because HIV is a relatively small category (Table 1), this only implies undercounting a small absolute number of pneumonia cases. On the other hand, a large percentage of other infections is misclassified by EAVA as pneumonia. Because other infections contribute a high percentage of deaths, this implies substantial overcounting of pneumonia cases. A net effect of these is overcounting for pneumonia, and hence the calibrated CSMFs for pneumonia is considerably lower than the uncalibrated CSMFs. Additionally, for EAVA we see a large increase in the CSMFs for other infections and decreases in CSMFs for diarrhea and HIV. A high percentage (28%) of deaths with MITS-COD as other infections were misclassified by EAVA as pneumonia, analogous to the misclassification rate for InSilicoVA. However, compared with InSilicoVA, EAVA has lower misclassification rates of deaths from other MITS causes being misclassified as other infections (especially for MITS severe malnutrition deaths). Hence, there is less overcounting and similar undercounting for other infections for EAVA compared with InSilicoVA, and we see that after calibration the EAVA CSMFs for “other infections” increases substantially.

The ensemble estimates are presented in Figure 3 (bottom left). The uncalibrated ensemble CSMF is simply an equally weighted average of the uncalibrated InSilicoVA and EAVA CSMF. The ensemble calibrated CSMF lies between the calibrated CSMF for InSilicoVA and EAVA, but the weights are cause specific and data driven. For malaria, the CSMF from ensemble calibration agrees more with the CSMF from calibrated InSilicoVA, which had much more certainty than the calibrated EAVA malaria CSMF, which had a wide credible interval. The final CSMF estimate from the ensemble is presented in the pie chart of Figure 3 (bottom right) and assigns 36% to other infections, 27% to malaria, 19% to diarrhea, and < 10% to each of the remaining causes. The 95% Bayesian credible intervals for the calibrated CSMF help assess for which set of causes the calibrated CSMF is substantially different from the uncalibrated ones. We see from Supplemental Table 1 that for the ensemble method, the 95% interval for calibrated CSMF for malaria (19–33%) lies above the uncalibrated estimate (14%), showing an increase in the CSMF after calibration. The interval for calibrated CSMF for pneumonia (5–12%) lies below the uncalibrated estimate (19%), showing a significant decrease. The uncalibrated CSMF for “other infections” is at the lower end of the interval for calibrated CSMF for this category (27–46%), showing an increase in CSMF for “other infections” after calibration.

**Figure 4.**
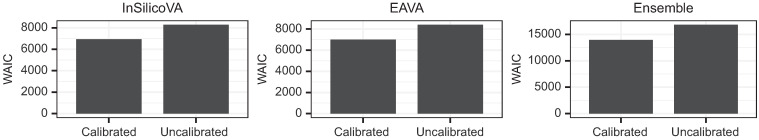
Model comparison for the uncalibrated and calibrated children (1–59 months) cause-specific mortality fractions estimates of InSilicoVA (left), EAVA (center), and Ensemble (right) using widely applicable information criterion (WAIC).

In Figure 4, we evaluate the uncalibrated and calibrated CSMF using WAIC for each of the three methods: InSilicoVA, EAVA, and ensemble (see Supplemental Section 2 for details of WAIC). The WAIC for the calibrated CSMF is consistently lower, offering evidence that the uncalibrated CSMF is incompatible with the observed misclassification rates and that adjustment via calibration substantially improves model fit for the combined COMSA and CHAMPS data.

### Neonatal results.

#### Raw VA summary statistics.

Among the 818 neonatal deaths, the analysis excludes VA records for 186 deaths for which the EAVA diagnoses were inconclusive. Table 2 presents these the VA-COD distributions for the remaining 632 COMSA neonate VA records. Both InSilicoVA and EAVA attribute most deaths (∼50%) to infection, with IPREs and prematurity being the two other major categories, each being attributed to ∼20–25% of the deaths. Both algorithms assigned < 5% of deaths to either congenital malformation or other.

**Table 2:** Raw counts and percentages of cause of death in neonates as predicted by InSilicoVA and EAVA for 632 COMSA neonate VA records

Algorithm	Congenital malformation	Infection	IPRE	Other	Prematurity
InSilicoVA, *n* (%)	1 (0.2)	290 (45.9)	161 (25.5)	12 (1.9)	168 (26.6)
EAVA, n (%)	28 (4.4)	333 (52.7)	132 (20.9)	21 (3.3)	118 (18.7)

COSMA = Countrywide Mortality Surveillance for Action; IPRE = intra-partum related event; VA = verbal autopsy.

#### CHAMPS MITS data and VA misclassification rate matrices.

The misclassification rates of the two CCVA algorithms for neonates were calculated based on CHAMPS/MITS data for neonatal deaths; 79 deaths were excluded from the analysis due to inconclusive EAVA diagnosis. Additionally, all the neonatal deaths from the CHAMPS site in South Africa were excluded due to a high proportion of nosocomial infections documented at the newborn intensive care unit. The signs and symptoms of the presenting illness, reported by the parents in the VA, may not correspond well to the illness causes by an infection acquired after hospitalization.

**Figure 5.**
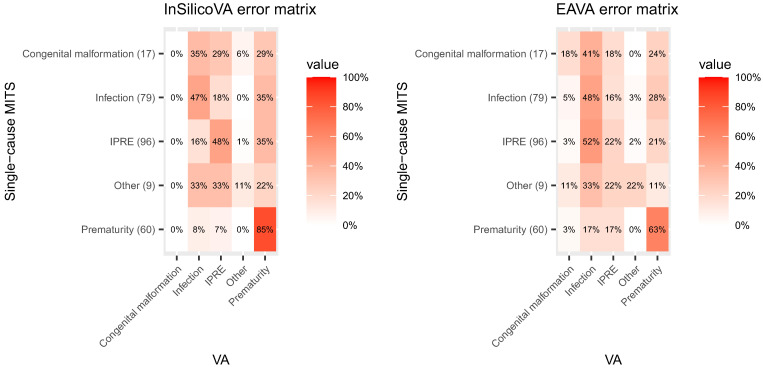
Misclassification rates matrices for InSilicoVA and EAVA for neonates based on the Child Health and Mortality Prevention Surveillance minimally invasive tissue sampling (MITS) data. The row totals indicate the counts for MITS underlying cause of death. IPRE = intra-partum related event; VA = verbal autopsy.

Figure 5 presents the misclassification rates. For InSilicoVA, prematurity has the highest sensitivity, with 85% of the MITS prematurity deaths correctly diagnosed by InSilicoVA. Infection and IPREs had moderate sensitivity (∼50%), whereas sensitivities for congenital malformation and other were low. There were also a few large misclassification rates, most prominent being InSilicoVA falsely diagnosing prematurity for ∼20–30% of deaths for each of the other four MITS causes. The misclassification rates for EAVA were broadly similar. The major difference was that the sensitivity of EAVA diagnosing prematurity was less (63%). Also, 52% of MITS IPRE deaths were misdiagnosed by EAVA as infection.

#### Calibrated CSMFs.

**Figure 6.**
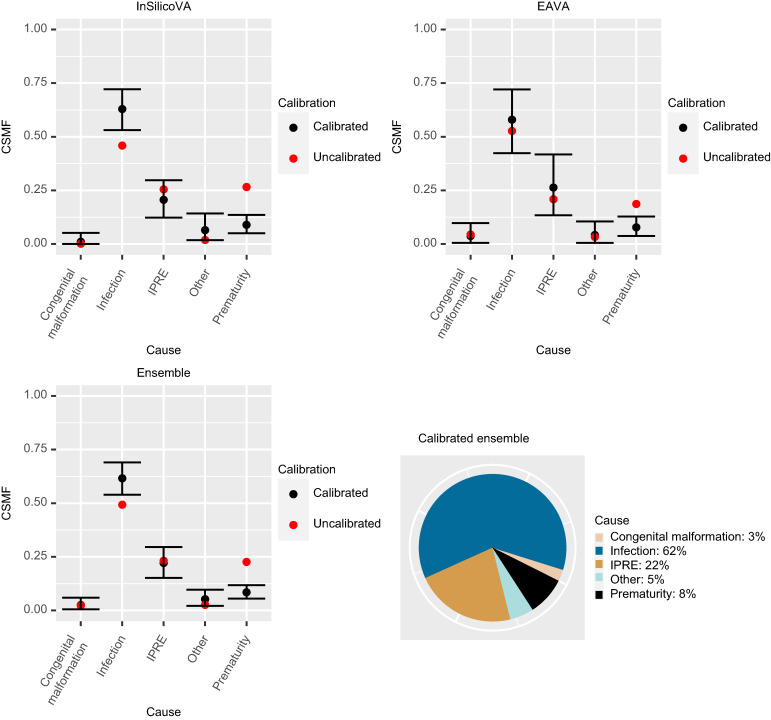
Calibrated cause-specific mortality fractions (CSMF) estimates for neonates for the three VA methods (top left: InSilicoVA; top right: EAVA; bottom left: Ensemble). The estimates (black points) are the posterior mean of the calibrated CSMFs. The black bars give the 95% credible intervals. For reference, the uncalibrated CSMF is also plotted (red-points). Bottom-right is the pie-chart of the point estimates (posterior means) of the calibrated ensemble CSMFs.

Figure 6 presents the calibrated CSMF point estimates and 95% credible intervals along with the uncalibrated CSMF for neonates. The impacts of calibration on the CSMFs are similar for all three methods: InSilicoVA, EAVA, and ensemble. The main changes after calibration are an increase in CSMFs for infection and a large decrease in CSMFs for prematurity. This is expected because a large proportion of infection deaths are misclassified as prematurity by both algorithms (Figure 5). For EAVA, the increase in CSMFs for infection is more moderate than for InSilicoVA. This is because, for EAVA, a considerable proportion of true IPRE and prematurity deaths are misdiagnosed as infection, so the calibration adjusts for it, and the gain in infection CSMFs by accounting for the misclassification of infection deaths as prematurity is partly offset by this adjustment.

The uncalibrated ensemble CSMF is simply the average of the uncalibrated InSilicoVA and EAVA CSMFs. The final estimate of CSMFs from the calibrated ensemble is presented in the bottom right of Figure 5. The calibrated ensemble attributes 62% of neonatal deaths to infection, 22% to IPREs, 8% to prematurity, and < 5% to each of congenital malformation and other. The exact numbers are provided in Supplemental Table 2. The 95% Bayesian credible intervals for the calibrated CSMF help assess for which set of causes the calibrated CSMF is substantially different from the uncalibrated ones. For the ensemble method, for infections the 95% interval for the calibrated CSMF (54–69%) lies above the uncalibrated CSMF for infection (49%), showing an increase in CSMF after calibration (Supplemental Table 2). For prematurity, the 95% interval for calibrated CSMF (6–12%) lies below the uncalibrated CSMF for prematurity (23%), showing a considerable decrease. For the other causes, the 95% interval for the calibrated CSMF covers the uncalibrated CSMF.

**Figure 7.**
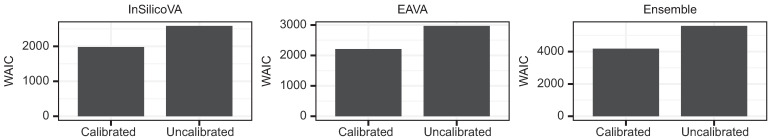
Model comparison for the uncalibrated and calibrated neonate cause-specific mortality fraction estimates of InSilicoVA (left), EAVA (center), and Ensemble (right) using widely applicable information criterion (WAIC).

We compare the performance of the uncalibrated and calibrated CSMFs for neonates using WAIC in Figure 7. Akin to the children results, the WAIC for the calibrated CSMFs is consistently and substantially better (lower) than the uncalibrated analogs. This demonstrates that the uncalibrated CSMFs do not provide an accurate description of the combined COMSA and CHAMPS data and that calibration is necessary to adjust for the large misclassification rates of the CCVA algorithms.

## DISCUSSION

This paper outlines the complete statistical workflow to use a limited dataset of paired VA records and a reference standard COD (in this case including results of MITS) to calibrate raw CSMF estimates obtained from CCVA algorithms applied to abundant VA data from a nationally representative survey (in this case COMSA). We show that for neonates and children age 1–59 months, and for two choices of CCVA algorithms, the COD predictions from CCVA have large misclassification rates. Naive estimates of CSMF that do not account for the misclassification will be biased, and calibration is necessary to mitigate this bias.

For child deaths in Mozambique, the calibration results in higher estimated mortality from malaria and other infections and lower estimated mortality from pneumonia. For neonates, the calibration results in increased CSMF for infection and decreased CSMF for prematurity. We provide insight into why the calibration resulted in these changes to the CSMF based on the misclassification rate matrices. However, in general, giving a simple explanation for the changes to the CSMF after calibration may not always be possible because the calibration reflects the total change affected by multiple different misclassification rates. This underscores the need for clear communication between statistical practitioners and government officials and stakeholders to understand the general principles of the calibration model, which are intuitive and interpretable.

The entire analysis in this manuscript used the top predicted COD from InSilicoVA. In practice, many probabilistic algorithms like InSilicoVA offer not just the most likely COD but probability scores for each cause to be the COD for an individual. Reducing such a rich probabilistic output to a single top cause wastes valuable information. Additionally, the analysis excluded some deaths because of indecisive EAVA diagnosis. Ideally this should be imputed to have the population proportion for each cause. Such imputed COD would also constitute a multi-cause COD output and cannot be accommodated in the single-cause format. Finally, MITS offers both an immediate and an underlying COD, and for many deaths these are different. The current analysis only used the underlying COD. An approach that accommodates multi-cause MITS output would be able to use information from both the underlying and the immediate COD.

Fiksel et al.^14^ extended the VA calibration method to accommodate multi-cause output for both the VA and the reference COD, based on the generalized definition of misclassification rates for such multi-output data.^14^^,^^29^ Subsequent work will apply this approach for calibration of CSMF based on multi-cause COMSA-VA and CHAMPS-MITS data. To use a multi-cause calibration for EAVA and also for the ensemble that uses EAVA, one innovation will be to apply a modified EAVA algorithm that offers multi-cause output as opposed to the EAVA algorithm used here, which only offers a single COD.

The calibration does not depend on the cause-specific composition of the MITS deaths, which is not representative of the population COD composition. Only the misclassification rates of VA for a given MITS cause are estimated from the CHAMPS data and used for calibration of COMSA data. There is a need to increase community MITS deaths for better representation of the VA misclassification rates. Hospital deaths, and especially NICU deaths, may even exhibit signs and symptoms not seen in community deaths due to effects of treatment, nosocomial infections, prolonged life, etc., and VA responses for these deaths may differ from those for community deaths in their exposure to health care and medical information. Also, the pooled CHAMPS data across all sites are used to improve sample size for estimation of the misclassification rates. This increased sample size is critical to improve precision of the analysis but may come with a loss of representativeness of the estimated misclassification rates for Mozambique. The impact of this tradeoff on the performance of the calibration needs to be quantitatively assessed. In the future, if more data are available on the VA-MITS pair for community deaths in Mozambique, the misclassification rates may be estimated solely using Mozambique CHAMPS data and may be more representative of these rates in the population.

Due to the limited sample size of the CHAMPS data, the calibration aggregated causes to a smaller set of broad cause categories (see “Aggregation of causes into broad categories” above) and produced calibrated CSMF at this broad resolution of causes. In the future, when more MITS data are available to estimate misclassification rates, these lists could be expanded to include more specific causes encompassed by these broad categories. For children, this would add to the current list injury and three neonatal causes that can still lead to death in the first year of life (i.e., prematurity, IPREs, and congenital malformation). For neonates, the infection category would be replaced by pneumonia, sepsis, meningitis, and other infections. With even more data, the child list could be further expanded to include causes such as specific injury types, childhood cancer, hemorrhagic fever, and other major conditions. The neonatal list could also include tetanus and injuries.

Despite these important unsolved challenges for producing calibrated CSMF estimates, given the large misclassification rates we observe for both VA algorithms, our method produces more informed CSMF estimates than simply aggregating VA algorithm predictions. The methodology adopted for calibration of COMSA CSMF using MITS offers a general template for calibration of VA-based CSMF in other studies. The calibratedVA software only requires as input the VA-COD for the unpaired data and both the VA-COD and the COD based on more extensive information (in our case, MITS-COD) for the paired data. The software works with any number of different CCVA algorithms (can be more than two) and with any type of reference COD in the paired data (e.g., a different COD based on the PHMRC data was used for VA calibration in Datta et al.^13^).

COD information is fundamental to prioritizing and planning disease control strategies and health services. VA is the major data source for COD information for 90% of child deaths globally^30^ and for all high-mortality countries. The use of calibration for nationally representative VA data can make these estimates more accurately reflect the true causes in a national population of children, better guiding national and international responses to reduce child deaths. As more countries begin implementing VAs within national mortality surveillance systems, there will be a need to obtain data on a smaller number of deaths with both VA-COD and some reference COD (like MITS-COD) based on more comprehensive information. This will inform the misclassification rates of VA for that country and, in turn, improve CSMF estimates via calibration. Projects such as the global symptom-cause archive^31^ may help to establish misclassification rates for many algorithms and regions of the world to produce accurate COD information for low- and middle-income countries.

## Financial Disclosure

Financial support: The COMSA Mozambique project is funded by the Bill & Melinda Gates Foundation (Grant no. OPP1163221).

## Supplemental Materials


Supplemental materials
