# Supplementary material for: Correcting for Verbal Autopsy Misclassification Bias in Cause-Specific Mortality Estimates
Source: Am J Trop Med Hyg. 2023 Apr 10;108(5 Suppl):66–77. doi: 10.4269/ajtmh.22-0318 (PMC10160858; doi:10.4269/ajtmh.22-0318)
Supplement: Supplementary file 1 [file tpmd220318.SD1.pdf]

Supplement to "Correcting for verbal autopsy misclassification bias in cause-specific mortality estimates of children and neonates in Mozambique"

S1. Technical details of the calibration

Let *M* denotes the mis-classification rate matrix of a CCVA algorithm, and *p* denote the vector of true CSMF that would be obtained by back-solving. The law of total probability postulates that the raw (uncalibrated) CSMF from the CCVA algorithm is given by $q=M'p$. Thus, the CCVA-predicted COD counts from the COMSA data can be modeled as Multinomial draws with probability q. This helps to estimate q. Similarly, the CCVA-predicted COD counts for the CHAMPS cases with MITS underlying cause *i* can be modeled as Multinomial draws with probability M_{i*}, the ith row of M. This allows estimation of M using the CHAMPS data. As $q=M'p$, the two pieces (estimates of *q* and *M*) are then combined to back-calculate and calibrate to obtain the true CSMF *p*. This is done jointly in a Bayesian framework that allows propagation of uncertainty of each of the estimate steps into the final estimates of *p* and ensures that *p* lies between 0 to 100%. The Bayesian implementation also offers the convenience of using shrinkage priors to stabilize the estimates. Shrinkage priors are important especially for low sample sizes of the paired data used to estimate the misclassification, as they help to stabilize (improve precision of) the estimates unlike direct back-solve. As recommended in [1], we use priors that shrink the calibrated estimate of *p* towards the uncalibrated estimate of *p* if there is not enough data to confidently estimate the misclassification rates.

S2. Model comparison using the WAIC

We have two collected sources and types of data which need to be modeled differently. The COMSA data correspond to a marginal multinomial likelihood for the CCVA predicted COD, and the CHAMPS data correspond to a conditional multinomial likelihood of the CCVA predicted COD given the MITS COD. If we just used the COMSA data, which has no other COD information (like MITS-COD), to evaluate the WAIC, the CCVA algorithms would not be penalized for their high degree of misclassification, as evidenced in the CHAMPS cases, and the best WAIC would be obtained from the uncalibrated model for the CSMF which is the best fit to the COMSA VA data if we ignore misclassification.

However, the uncalibrated CSMF assumes that the models have perfect sensitivity and the CHAMPS data testifies for or against this assumption. Hence, the misclassification rates are critical to understand the relationship between the VA-COD and the MITS-COD, and the WAIC calculation needs to include the CHAMPS data as well. Thus in our case, the future data for WAIC are twofold – VA-COD predictions for nationally representative community deaths

(COMSA data), which are modeled using both the true CSMF and misclassification rates, and VA-COD predictions for the CHAMPS data with MITS-COD provided, which are modeled using just the misclassification rates. If the CHAMPS data demonstrate large misclassification rates, the WAIC will be large thereby rightfully penalize the uncalibrated CSMF for the wrong assumption of perfect sensitivity.

To estimate the WAIC from the calibrated models, we use the MCMC draws of the CSMF and the misclassification rates to obtain the posterior distribution of the log-likelihood for every death in both sources of data.

To estimate the WAIC for the uncalibrated models, we first obtain draws from the posterior distribution of the CSMF by assuming perfect sensitivity. The posterior mean of this distribution is nearly exactly that of the uncalibrated CSMF estimate. The prefect sensitivity assumption for the uncalibrated model, ideally translates to all posterior draws of the misclassification matrix being the identity matrix. This however produces a WAIC of infinity immediately ruling out the uncalibrated model. To make the WAIC of the uncalibrated model more competitive to that of the calibrated model, we compute the former assuming model sensitivities of 95%, under the assumption that with sufficiently high (but not perfect) sensitivity, one would still be willing to accept the uncalibrated CSMF estimates.

S3. Implementation of CCVA algorithms

To run InSilicoVA, the data variables and values are converted from the VA questionnaire format to the openVA R package format. For example, the variable Id10019, which is sex in the VA questionnaire, is recoded i019a and i019b and response values for i019a are “Y” if male and “N” if female, and vice-versa for i019b. Missing, don’t know, and refusal are coded “.” This mapping process, for each of the hundreds of VA questionnaire variables, must be done manually and checked separately, for each data source. For InSilicoVA inputs we assume the prevalence of HIV and malaria to be high in Mozambique. To run EAVA, the VA questionnaire variables and responses remain in the same format as they were collected. The EAVA CCVA creates a database indicating a diagnosis is either present or absent, based on reported symptoms. Causes are then assigned for each age group based on a hierarchy.

S4. Uncalibrated and calibrated CSMFs

Table S1: Raw (uncalibrated) and calibrated CSMF estimates (along with 95% confidence intervals) for children (1-59 months) from the 3 VA methods.

		Malaria	Pneumonia	Diarrhea	Severe malnutrition	HIV	Other	Other infections
InSilicoVA	Uncalibrated	19%	16%	25%	5%	4%	8%	24%
	Calibrated	28% (22% - 34%)	7% (3% - 12%)	26% (19% - 33%)	6% (1% - 16%)	6% (1% - 12%)	8% (2% - 14%)	19% (10% - 31%)
EAVA	Uncalibrated	8%	22%	19%	6%	8%	6%	30%
	Calibrated	19% (5% - 36%)	9% (5% - 16%)	12% (6% - 19%)	4% (1% - 9%)	2% (0% - 4%)	3% (1% - 7%)	51% (33% - 67%)
Ensemble	Uncalibrated	14%	19%	22%	5%	6%	7%	27%
	Calibrated	27% (19% - 33%)	8% (5% - 12%)	19% (14% - 25%)	4% (1% - 8%)	2% (1% - 4%)	4% (2% - 8%)	36% (27% - 46%)

Table S2: Raw (uncalibrated) and calibrated CSMF estimates (along with 95% confidence intervals) for neonates from the 3 VA methods.

		Congenital malformation	Infection	IPRE	Other	Prematurity
InSilicoVA	Uncalibrated	0%	46%	25%	2%	27%
	Calibrated	1% (0% - 5%)	63% (53% - 72%)	21% (12% - 30%)	6% (2% - 14%)	9% (5% - 14%)
EAVA	Uncalibrated	4%	53%	21%	3%	19%
	Calibrated	4% (1% - 10%)	58% (42% - 72%)	26% (13% - 42%)	4% (1% - 11%)	8% (4% - 13%)
Ensemble	Uncalibrated	2%	49%	23%	3%	23%
	Calibrated	3% (1% - 6%)	62% (54% - 69%)	22% (15% - 30%)	5% (2% - 10%)	8% (6% - 12%)

[1] A. Datta, J. Fiksel, A. Amouzou, and S. L. Zeger, “Regularized Bayesian transfer learning for population-level etiological distributions,” *Biostatistics*, Feb. 2020.
